# Treatment costs for patients with *Streptococcus suis* infection in Northern Thailand: a hospital-based observational study of 14-year data

**DOI:** 10.1186/s12889-023-15623-w

**Published:** 2023-04-21

**Authors:** Ajaree Rayanakorn, Wasan Katip, Zanfina Ademi, Kok-Gan Chan

**Affiliations:** 1 Faculty of Public Health, Chiang Mai University, 239 Huay Kaew Road, Tambon Suthep, Muang District, Chiang Mai, 50200 Thailand; 2 School of Pharmacy, Monash University Malaysia, Jalan Lagoon Selatan, Selangor 47500 Bandar Sunway, Malaysia; 3 Department of Pharmaceutical Care, Faculty of Pharmacy, Chiang Mai University, Chiang Mai, Thailand; 4 Centre for Medicine Use and Safety, Faculty of Pharmacy and Pharmaceutical Sciences, Monash University, VIC 3052 Melbourne, Australia; 5 School of Public Health and Preventive Medicine, Monash University, Melbourne, Australia; 6 Division of Genetics and Molecular Biology, Faculty of Science, Institute of Biological Sciences, University of Malaya, 50603 Kuala Lumpur, Malaysia; 7 International Genome Centre, Jiangsu University, Zhenjiang, China

**Keywords:** *Streptococcus suis*, *S.suis*, Cost of illness, Direct medical cost, Thailand

## Abstract

**Background:**

*Streptococcus suis (S.suis)* is a neglected zoonotic disease that imposes a significant economic burden on healthcare and society. To our knowledge, studies estimating the cost of illness associated with *S.suis* treatment are limited, and no study focuses on treatment costs and potential key drivers in Thailand. This study aimed to estimate the direct medical costs associated with *S.suis* treatment in Thailand and identify key drivers affecting high treatment costs from the provider’s perspective.

**Methods:**

A retrospective analysis of the 14-year data from 2005–2018 of confirmed *S.suis* patients admitted at Chiang Mai University Hospital (CMUH) was conducted. Descriptive statistics were used to summarize the data of patients’ characteristics, healthcare utilization and costs. The multiple imputation with predictive mean matching strategy was employed to deal with missing Glasgow Coma Scale (GCS) data. Generalized linear models (GLMs) were used to forecast costs model and identify determinants of costs associated with *S.suis* treatment. The modified Park test was adopted to determine the appropriate family. All costs were inflated applying the consumer price index for medical care and presented to the year 2019.

**Results:**

Among 130 *S.suis* patients, the average total direct medical cost was 12,4675 Thai baht (THB) (US$ 4,016), of which the majority of expenses were from the “others” category (room charges, staff services and medical devices). Infective endocarditis (IE), GCS, length of stay, and bicarbonate level were significant predictors associated with high total treatment costs. Overall, marginal increases in IE and length of stay were significantly associated with increases in the total costs (standard error) by 132,443 THB (39,638 THB) and 5,490 THB (1,715 THB), respectively. In contrast, increases in GCS and bicarbonate levels were associated with decreases in the total costs (standard error) by 13,118 THB (5,026 THB) and 7,497 THB (3,430 THB), respectively.

**Conclusions:**

IE, GCS, length of stay, and bicarbonate level were significant cost drivers associated with direct medical costs. Patients’ clinical status during admission significantly impacts the outcomes and total treatment costs. Early diagnosis and timely treatment were paramount to alleviate long-term complications and high healthcare expenditures.

**Supplementary Information:**

The online version contains supplementary material available at 10.1186/s12889-023-15623-w.

## Background

*Streptococcus suis (S.suis)* is a gram-positive alpha hemolytic bacterium mainly in pigs which can cause severe infection in humans through exposure or contact with pigs and consumption of raw pork [1]. *S.suis* infection has been predominantly found among working-age men and imposed substantial health and economic burden, particularly in Southeast Asia [1–4]. The highest cumulative disease prevalence is in Thailand (8.21 cases/million population), accounting for 11% of all cases reported worldwide [4, 5]. The clinical manifestations include meningitis, septicaemia, infective endocarditis (IE), resulting in death and long-term complications among survivors [6–8].

The disease has been notable, causing substantial losses in the swine industry [9]. In humans, it has been estimated to cause disability-adjusted life years (DALYs) loss ranging from 1,437 to 1,866 and the direct medical cost of US$1,635 per episode in Vietnam [2]. According to a recent study in Thailand, *S.suis* infection incurred 769 years of life lost (YLL), 826 quality-adjusted life years (QALYs) lost, and 793 productivity-adjusted life years (PALYs) lost in 2019, which was associated with a loss of US$11.3 million in GDP or US$36,033 lost per person [3]. This reflects significant economic impact despite the disease's perceived low prevalence, partly due to being neglected and underreported.

To our knowledge, studies estimating treatment costs associated with *S.suis* treatment are still limited. Up to date, no study has investigated the cost of illness from *S.suis* treatment and factors affecting the treatment costs in Thailand. Therefore, this study aimed to estimate the real-world financial burden concerning direct medical costs associated with *S.suis* treatment in Thailand and identify key drivers affecting high treatment costs from the healthcare provider’s perspective.

## Methods

### Study design and data collection

This was a cross-sectional study of the 14-year data at Chiang Mai University Hospital (CMUH), a 1400-bed tertiary teaching hospital during 2005–2018, the largest hospital in northern Thailand. The hospital’s electronic database was used to retrieve healthcare utilisation of *S.suis* positive patients admitted from January 2005 to December 2018 with confirmed positive *S.suis* either by cerebrospinal fluid (CSF) or hemoculture. The index date was the hospital admission date. *S.suis* cases with specimens taken from CSF or hemoculture were identified from the hospital laboratory records of microbiological proven *S.suis* patients with their hospital numbers (HNs). All confirmed *S.suis* cases with complete medical and healthcare resource utilization records who had been admitted from 1 May 2005 to 31 December 2018 were collected and reviewed. All costs were derived from hospital charges provided by the Office of Medical Records and Statistics, Chiang Mai University Hospital, to estimate the direct medical costs associated with treatment during the episode.

The study was approved by the Research Ethics Committee, Faculty of Medicine, Chiang Mai University (IRB no.010/2018) and Monash University Human Research Ethics Committee (MUHREC) (Project no.12225). Patients’ names, and personal and traceable information were omitted and treated as confidential in all study procedures. The informed consent was waived due to the retrospective nature of the study.

### Cost estimation

This study considered the healthcare provider’s perspective and included only direct medical costs associated with *S.suis* treatment which is generally fully paid by the provider in Thailand’s healthcare system. Direct medical costs included medication, laboratory tests, and others, e.g., room charges, meals, medical staff services, and medical devices during hospital admission. All costs were inflated to the 2019 value using Consumer Price Index with the medical care section [10]. The average market exchange rate in 2019 was US$ = 31.0470 Thai baht (THB) [11].

### Statistical analysis

Descriptive statistics were used to summarize the data on patients’ characteristics, healthcare utilization and costs. Multiple imputations with predictive mean matching on log-transformed costs were employed to deal with missing data [12] which was Glasgow Coma Score (GCS) data. Due to the skewness of the cost data, Generalized linear models (GLMs) were used to forecast costs models and identify determinants of costs [13] associated with *S.suis* treatment. In addition, the models had the advantage of displaying both the mean and variance functions directly based on the original scale. Therefore, there would not be any issue concerning retransformation. Potential multicollinearity was examined before building the final model. The modified Parks test was adopted to determine the appropriate family for healthcare expenditures based on the relationship between raw scale means and variance function [13]. The interpretation for family was based on the coefficients for lnyhat: 0 Gaussian test lnyhat = 0, 1 Poisson test lnyhat = 1, 2 Gamma test lnyhat = 2, 3 Inverse Gaussian or Wald test lnyhat = 3 [14]. After the model development, the bootstrapping technique was applied to estimate bootstrap-corrected calibration coefficients for internal validation [15]. Significant predictors and costs were illustrated as marginal estimates for ease of interpretation. All analyses were executed using Stata/IC version 16.0 for Windows (StataCorp LP, College Station, TX, USA).

## Results

### Patients’ characteristics

A total of 130 confirmed *S.suis* patients with complete medical records and hospital charges data were eligible based on the inclusion criteria. Among these, there were 89 males and 41 females. However, Glasgow Coma Score (GCS) data and length of stay were available only among 101 and 128 patients respectively. The mean age was 56.32 ± 13.77 years. The significant risk behaviors included alcohol drinking (50.77%) and eating raw pork (37.69%). Septicaemia was the most common clinical presentation (56.15%), followed by meningitis (36.92%) and infective endocarditis (IE) (26.15%) (Table 1). Approximately half of *S. suis* infected patients recovered from their infection (52.31%), while around one-third recovered with sequelae (33.08%). The average admission duration was 18 days. The characteristics of *S.suis* patients are presented in Table 1.

**Table 1:** Patient characteristics

Characteristics	Total (*n* = 130)
**Age (year) (mean ± SD)**	56.32 ± 13.77
**Male**	89 (68.46%)
**Female**	41 (31.54%)
**GCS**^**a**^	12.65 ± 3.15
**SAPS II**	27.22 ± 13.93
**Risk behaviours**	
- Alcohol drinking	66 (50.77%)
- Consumption of raw pork	49 (37.69%)
- Recent contact with pigs/pork exposure	5 (3.85%)
- Pig related occupation	3 (2.31%)
- Skin injury	2 (1.54%)
**Signs and symptoms**	
- Neck stiffness	46 (35.38%)
- Diarrhea	37 (28.46%)
- Vomiting	27 (20.77%)
- Vertigo	10 (7.69%)
**Major clinical manifestations**	
- Septicemia	73 (56.15%)
- Meningitis	48 (36.92%)
- IE	34 (26.15%)
- Septic shock	20 (15.38%)
**Outcomes**	
- Recovered	68 (52.31%)
- Recovered with sequelae	43 (33.08%)
- Having valve replacement	16 (12.31%)
- Death	16 (12.31%)
**Mean length of stay (days)**^**b**^	18.27 ± 17.26

*GCS* Glasgow coma score, *IE* Infective endocarditis, *SAPS II* The Simplified Acute Physiology Score

^a^Available data from 101 patients

^b^Available data from 128 patients

### Costs of *S.suis* treatment

The cost data was right skewed with a long heavy right tail reflecting substantial more services required among severe cases and higher costs in relatively small number of patients (See Figure S1 in Online supplementary material). Total treatment cost of *S.suis* was estimated to be 16,207,676 (US$ 522,037.12) in 2019 (Table 2). The mean cost of medications, laboratory tests, X-ray, and others (room charges, meals, staff services, medical devices) for *S.suis* treatment paid by the hospital in 2019 value were 30,322 (US$ 977), 13,187 (US$ 425), 8,240 (US$ 265), and 72,926 (US$ 2,349), respectively. Majority of expenses were from “others” category. The average total treatment cost per episode was 124,675 (US$ 4,016). After removing patients who died at the hospital and rerunning the analysis, the similar trends were observed while the mean length of stay was 19.25 (SD = 17.62) days (See Tables S1 and S2 in Online supplementary material).

**Table 2:** Direct medical cost of *Streptococcus suis* treatment paid by the hospital (2019 value prices)

Cost variables	Mean	SD	Median	IQR
Overall (*n* = 130)				
Medications	30,321.75 (US$ 976.64)	61,930.05	8,132.181	4,223.40–23,588.58
Laboratory tests	13,187.22 (US$ 424.75)	14,195.96	8,087.821	4,820.37–16,670.44
X-ray	8,239.51 (US$ 265.39)	12,487.7	4,039.29	714–9,828
Others (Room charges, meals, staff services, medical devices)	72,926.03 (US$ 2,348.89)	98,299.8	24,231.56	94,65.50–99,540.23
**Total cost**	16,207,676.40 (US$ 522,037.12)	168,825.40	47,213.39	25,136.02–182,052.60

### Key drivers affecting high treatment costs

From univariable analysis, there were 15 significant clinical variables associated with total treatment cost, including corticosteroid use, sensorineural hearing loss (SNHL), valvular heart disease (VHD), recent pigs/pork exposure, having sequelae, GCS, SAPS, meningitis, IE, vomiting, neck stiffness, length of stay, bicarbonate level, serum phosphorus, blood urea nitrogen. After multicollinearity checking, two predictors were removed: VHD and neck stiffness. According to multivariable GLM analyses for costs employing stepwise forward logistic regression at *p*-value < 0.05, four predictors remained in the final model: IE, GCS, bicarbonate level and length of stay.

A bootstrap of 1000 sampling with replacement was performed for interval validation. The bootstrap-corrected calibration coefficient of the total cost model was 0.716 with a mean optimism equating to -0.0057 (95%CI -0.202 to 0.289), reflecting low bias and a good model.

Infective endocarditis (IE), GCS, length of hospital stay, and bicarbonate were associated with the high total treatment costs of *S.suis* infection (Table 3). Overall, marginal increases in IE and length of stay were strongly associated with total cost (standard error) increases of 132,443 THB (39,638 THB) and 5,490 THB (1,715 THB), respectively. In contrast, increases in GCS and bicarbonate level were related to total cost (standard error) decreases of 13,118 THB (5,026 THB) and 7,497 THB (3,430 THB), respectively. The average marginal estimates are presented in Table 4.

**Table 3:** Cost Model of *Streptococcus suis* treatment

Variables	Unstandardised Coefficients		t	Significant level	95% Confidence Interval for B	
	**Beta**^**a**^	**SE**^**a**^			**Lower Bound**	**Upper Bound**
Constant	333637.8	164,836.2	25.74	< 0.001	126687.2	878653.4
Infective Endocarditis	2.417703	0.4773245	4.47	< 0.001	1.641917	3.560039
GCS	0.9162752	0.0259905	-3.08	0.002	0.8667249	0.9686582
Length of stay	1.037278	0.0064089	5.92	< 0.001	1.024792	1.049915
Bicarbonate (mmol per litre)	0.9512553	0.0195557	-2.43	0.015	0.9136888	0.9903663

^a^Estimates were derived from the multivariable generalized linear model with gamma distribution and log link after the modified Park’s test

**Table 4:** Average marginal estimates

Variables	Delta-method		t	Significant level	95% Confidence Interval for B	
	**Dy/dx**	**SE**			**Lower Bound**	**Upper Bound**
Infective Endocarditis	132,442.7	39638.1	3.34	0.001	54753.47	210132
GCS	-13117.77	5026.173	-2.61	0.009	-22968.88	-3266.649
Length of stay	5490.756	1715.418	3.20	0.001	2128.598	8852.915
Bicarbonate (mmol per litre)	-7497.059	3430.863	-2.19	0.029	-14221.43	-772.6909

*GCS* Glasgow coma score

## Discussion

The study estimated direct medical costs associated with *S.suis* treatment among admitted infected patients for a single episode under the healthcare provider’s perspective at the largest university-affiliated hospital in northern Thailand. The average total treatment cost per episode was 12,4675 Thai baht (THB), or $4,016, with the majority of expenses falling under the "others" category (room and board costs, personnel services, and medical equipment). According to GLM multivariable analyses, IE, GCS, length of stay, and bicarbonate level were significant predictors of high treatment costs. Overall, marginal increases in IE and length of stay were significantly associated with increases in the total costs. Conversely, marginal gains in GCS and bicarbonate levels were significantly associated with decreases in the total costs.

Most patients were male, who are usually the family’s primary breadwinner while the total hospital treatment cost per episode (US$4,016) is more than half of the average annual income per capita (US$7,814 in 2019) [16]. The cost per episode in this study was higher than the previous finding reported in Vietnam (US$1,635 per episode) [2]. This reflects different healthcare costs, year of estimation, major clinical manifestations, and costs between studies. The higher hospital expenses found in our analysis imply the severity of the disease even during the acute stage.

The median length of stay among infected patients in the study was 18 days which was consistent with the average reported in previous literature (17 and 23 days) [4, 8] The relatively long length of stay might have been driven by major clinical manifestations, including meningitis, septicaemia, and IE. Septicaemia was usually concomitantly with meningitis or IE and a prominent cause of other complications, including septic shock, Disseminated Intravascular Coagulation (DIC), and associated multi-organ failure [6]. These could have culminated in extended hospitalization resulting in high expenses in “the others” cost category, which was the major cost component.

Meningitis is a major clinical presentation accounting for more than one-third of our *S.suis* patients. This condition usually requires extended hospitalization and leads to long-term sequelae, particularly hearing loss incurring high healthcare expenditure. The benefits of adjunctive corticosteroids in combination with antibiotic therapy in reducing the risk of hearing loss among adults with acute bacterial meningitis was demonstrated in a randomised controlled trial, and a network meta-analysis [17, 18]. However, a meta-analysis concluded that corticosteroids use was associated with reduced hearing and neurological complications only among acute bacterial meningitis patients in high-income countries without benefits for patients from low-income countries [19]. Despite inconsistent findings being noted [20, 21], none of the regimen containing corticosteroids was associated with harm [17]. Therefore, using adjunctive corticosteroids seems worthwhile considering the high risk of hearing impairment among *S.suis* meningitis patients. In this study, meningitis and corticosteroids use were significant in univariable analyses but not significant in the multivariable analyses. This might be due to smaller sample size of *S.suis* patients in our study.

Based on our findings, the increase in GCS and a higher level of serum bicarbonate were associated with lower treatment costs. This suggests that patients in stable condition are likely to benefit from treatment with more favourable outcomes at a lower cost. Low serum bicarbonate level was related to multiple detrimental manifestations, including progression of chronic kidney disease, metabolic acidosis, and all-cause mortality [22–24]. These deleterious complications would potentially increase healthcare resource utilisation and total treatment cost. Clinically, giving bicarbonate to patients may be considered in some circumstances such as to balance acid–base condition when patient’s PH level is at critical level. In our previous study, bicarbonate < 18 mmol/L was a significant predictor of mortality in univariate analysis [6]. However, it was not significant in the final model. Therefore, this should be done with caution and further studies with larger sample size are warranted before providing definite recommendations.

Some limitations can be noted in our analyses. Only the treatment costs during the first episode of admitted *S.suis* patients were analysed. However, *S.suis* is a severe disease which usually requires hospitalisation and immediate medical attention. Therefore, direct medical costs associated with the treatment should have been captured. Due to the retrospective nature of the study, there might be potential recalled bias relating to information on risk behaviors and exposure to pigs/raw pork. We did not include long-term treatment costs from complications, mainly IE, hearing impairment, and vestibular dysfunction, which could incur tremendous long-term costs. Additionally, the direct cost estimates were based on data from a university-affiliated tertiary hospital which may not reflect healthcare costs at lower-level setting. Finally, the healthcare provider’s perspective used in the study limited frame of costs included while direct non-medical costs, productivity costs, and out-of-pocket costs were not captured. Further studies considering societal aspects are warranted to provide a clearer picture of *S.suis* cost of illness and its consequences in society.

## Conclusions

In conclusion, this study provides consolidating evidence on *S.suis* cost of illness and key drivers. The analyses demonstrate substantial hospitalisation costs incurred from *S.suis* infection, of which the majority of cases are middle-aged men who are usually the main income generator of families in Thai society. This suggests potential catastrophic medical costs upon acute infection among affected families. According to the cost model in our study, IE, GCS, length of stay, and bicarbonate level were significant predictors associated with high total treatment costs. Patients’ clinical stability, especially GCS and bicarbonate level at admission, are crucial to the outcomes and healthcare costs. This emphasises the importance of timely diagnosis and early treatment in averting high direct medical costs and complications.

## Supplementary Information

**Additional file 1. **Online Supplementary Material.

## Data Availability

Additional file 1. Online Supplementary Material.
