# Supplementary material for: Treatment costs for patients with Streptococcus suis infection in Northern Thailand: a hospital-based observational study of 14-year data
Source: BMC Public Health. 2023 Apr 21;23:737. doi: 10.1186/s12889-023-15623-w (PMC10120222; doi:10.1186/s12889-023-15623-w)
Supplement: Supplementary file 1 — Additional file 1. Online Supplementary Material. [file 12889_2023_15623_MOESM1_ESM.docx]

**Treatment Costs for Patients with *Streptococcus suis* Infection in Northern Thailand: A Hospital-based Observational Study of 14-year Data**

Ajaree Rayanakorn, Wasan Katip, Zanfina Ademi, Kok-Gan Chan

**Online Supplementary Material**

[Figure S1 Overall distribution of cost data 2](#_Toc1389044)

The Modified Park Test [3](#_Toc1389045)

[Table S1. Direct medical cost of *S.suis* treatment paid by the hospital after excluding dead patients in the year 2019 value](#_Toc1389046) 5

Table S2. Cost Model of *S.suis* treatment after removing dead patients………………………6 [Table S3 Univariable analysis of potential predictors associated with *S.suis* total treatment cost 7](#_Toc1389048)

# **Overall distribution of cost data**

**Figure S1** Overall distribution of cost data

totalcostus2019

-------------------------------------------------------------

Percentiles Smallest

1% 269.2695 150.0515

5% 360.5148 269.2695

10% 539.2585 270.8514 Obs 130

25% 799.287 274.5464 Sum of Wgt. 130

50% 1501.313 Mean 3964.459

Largest Std. Dev. 5368.389

75% 5788.993 19079.72

90% 10222.88 23185.1 Variance 2.88e+07

95% 12874.01 25648.14 Skewness 2.665387

99% 25648.14 33823.91 Kurtosis 12.0516

# **The Modified Park Test**

The Modified Parks test was implemented after GLM regression that used the particular link to recommend a family. The test predicts the square of the rediduals (res2) as a function of the log of the predictions (lnyhat) according to the interpretation below [1]:

**eTable 1 Interpreting the coefficients from the modified park test**

Coefficient Family Stata® test

0 Gaussian test lnyhat = 0

1 Poisson test lnyhat = 1

2 Gamma test lnyhat = 2

3 Inverse Gaussian or Wald test lnyhat = 3

The stata code: glm y x, link(linkname) family (family name) was used to identify appropriate family. According to the analysis the redidual (res2) was close to 2 which indicated a Gamma family.

| Robust

r2_totalcostus2019 | Coef. Std. Err. z P>|z| [95% Conf. Interval]

-----------------------+----------------------------------------------------------------

lnyhat_totalcostus2019 | 2.447082 .4207034 5.82 0.000 1.622519 3.271646

_cons | -3.91184 3.227983 -1.21 0.226 -10.23857 2.414891

According to the test to confirm the recommended family for lnyhat = 2, the probability > chi2 was 0.2879 which also implied gamma family.

The details of Stata output was as follows:

test lnyhat ==0

( 1) [r2_totalcostus2019]lnyhat_totalcostus2019 = 0

chi2( 1) = 33.83

Prob > chi2 = 0.0000

. test lnyhat==1

( 1) [r2_totalcostus2019]lnyhat_totalcostus2019 = 1

chi2( 1) = 11.83

Prob > chi2 = 0.0006

. test lnyhat==2

( 1) [r2_totalcostus2019]lnyhat_totalcostus2019 = 2

chi2( 1) = 1.13

Prob > chi2 = 0.2879

. test lnyhat==3

( 1) [r2_totalcostus2019]lnyhat_totalcostus2019 = 3

chi2( 1) = 1.73

Prob > chi2 = 0.1888

# **Analysis after removing dead patients.**

**Table S1 Direct medical cost of *Streptococcus suis* treatment paid by the hospital after excluding dead patients (2019 value prices)**

| Cost variables | Mean | SD | Median | IQR |
| --- | --- | --- | --- | --- |
| Overall (n=114) |  |  |  |  |
| Medications | 27164.36  (US$ 874.94) | 46096.92 | 7373.03 | 4126.32- 22888.77 |
| Laboratory tests | 12873.54  (US$ 414.65) | 13620.11 | 7761.03 | 4,809.23-16,670.44 |
| X-ray | 8425.46  (US$ 271.38) | 13091.51 | 4,016.165 | 714.00-9,806.44 |
| Others (Room charges, meals, staff services, medical devices) | 73,204.96  (US$ 2,357.87) | 97334.67 | 24231.56 | 9266.89-124692.70 |
| Average cost per episode | 121,667.4 (US$ 4,015.67) | 154458 | 46312.63 | 22989.78-186382.6 |
| Total | 13,870,085.70 (US$ 446,744.8) |  |  |  |

**Table S2 Cost Model of *Streptococcus suis* treatment after removing dead patients**

| Variables | Delta-method | | t | Significant level | 95% Confidence Interval for B | |
| --- | --- | --- | --- | --- | --- | --- |
|  | **Dy/dx** | **SE** |  |  | **Lower Bound** | **Upper Bound** |
| Infective Endocarditis | 154620.10 | 37785.40 | 4.09 | < 0.001 | 80562.05 | 228678 |
| GCS | -10510.86 | 4406.11 | -2.39 | 0.017 | -19146.67 | -1875.05 |
| Length of stay | 5445.53 | 1486.15 | 3.66 | < 0.001 | 2532.74 | 8358.32 |
| Bicarbonate (mmol per litre) | -4908.01 | 2979.89 | -1.65 | 0.100 | -10748.48 | 932.46 |

GCS, Glasgow coma score

# **Univariable analysis of variables**

**Table S3 Univariable analysis of potential predictors associated with *S.suis* total treatment cost**

| Predictor variables | Coefficient* | SE* | P-value | 95%CI | |
| --- | --- | --- | --- | --- | --- |
|  |  |  |  | Lower Bound | Upper Bound |
| Age | 0.013 | 0.009 | 0.139 | -0.004 | 0.029 |
| Sex | -0.329 | 0.241 | 0.172 | -0.801 | 0.144 |
| GCS | -0.095 | 0.038 | 0.011 | -0.169 | 0.022 |
| SAPS II | 0.019 | 0.008 | 0.019 | 0.003 | 0.035 |
| VHD | 0.810 | 0.270 | 0.003 | 0.281 | 1.340 |
| Risk behaviours |  |  |  |  |  |
| - Alcohol drinking | 0.346 | 0.243 | 0.155 | -0.131 | 0.822 |
| - Raw pork consumption | -0.105 | 0.250 | 0.676 | -0.596 | 0.386 |
| - Recent contact with pigs/pork exposure | -1.888 | 0.596 | 0.002 | -3.055 | - 0.720 |
| - Pig related occupation | 0.157 | 0.796 | 0.844 | -1.403 | 1.717 |
| - Skin injury | 0.970 | 0.983 | 0.324 | -0.965 | 2.896 |
| Signs and symptoms |  |  |  |  |  |
| - Neck stiffness | -0.778 | 0.245 | 0.001 | -1.257 | -0.298 |
| - Diarrhea | 0.101 | 0.263 | 0.700 | -0.415 | 0.617 |
| - Vomiting | -0.893 | 0.280 | 0.001 | -1.442 | -0.345 |
| - Vertigo | -0.413 | 0.446 | 0.355 | -1.287 | 0.462 |
| Major clinical manifestations |  |  |  |  |  |
| - Septicemia | 0.062 | 0.238 | 0.795 | -0.405 | 0.529 |
| - Meningitis | -0.903 | 0.240 | < 0.001 | -1.373 | -0.434 |
| - IE | 1.032 | 0.294 | < 0.001 | 0.457 | 1.609 |
| - Septic shock | 0.391 | 0.324 | 0.227 | -0.244 | 1.026 |
| - Death | 0.182 | 0.356 | 0.608 | -0.515 | 0.881 |
| Corticosteroid use | -0.999 | 0.273 | < 0.001 | -1.535 | -0.463 |
| Presence of any sequelae | -1.040 | 0.228 | < 0.001 | -1.486 | -0.593 |
| SNHL | -1.159 | 0.219 | < 0.001 | -1.589 | -0.729 |
| Bicarbonate level | -0.087 | 0.028 | 0.002 | -0.143 | -0.032 |
| Serum phosphorus | 0.222 | 0.086 | 0.010 | 0.054 | 0.391 |
| Albumin | -0.105 | 0.221 | 0.635 | -0.537 | 0.328 |
| BUN | 0.013 | 0.006 | 0.015 | 0.003 | 0.024 |
| Mean length of stay | 0.046 | 0.009 | < 0.001 | 0.028 | 0.063 |

***Note:* ***Estimates were derived from the generalized linear model with gamma distribution and log link; BUN, Blood urea nitrogen; GCS, Glasgow coma score; IE, Infective endocarditis; SAPS II, The Simplified Acute Physiology Score; SNHL, Sensorineural hearing loss; VHD, Valvular heart disease

The following predictor variables showed multicollinearity:

- Valvular heart disease and endocarditis (corr = 0.758)
- Meningitis and neck stiffness (corr = 0.867)

Therefore, either one of the predictors was selected in the final model.

**References**

1. Glick DHA, Doshi DJA, Sonnad DSS, Polsky DD. 96Analyzing cost. 2014 [cited 2/9/2023]. In: Economic Evaluation in Clinical Trials [Internet]. Oxford University Press, [cited 2/9/2023]; [0]. Available from: https://doi.org/10.1093/med/9780199685028.003.0005.
